# Self-directed behavior reflects social stress in captive Asian elephants

**DOI:** 10.3389/fvets.2025.1629664

**Published:** 2025-06-13

**Authors:** Sofia Vilela, Peini Chen, So Murakami, Yusuke Tanabe, Shinya Yamamoto

**Affiliations:** ^1^Graduate School of Science, Kyoto University, Kyoto, Japan; ^2^Faculty of Science, Kyoto University, Kyoto, Japan; ^3^Faculty of Agriculture, Kyoto University, Kyoto, Japan; ^4^Institute for the Future of Human Society, Kyoto University, Kyoto, Japan

**Keywords:** aggression, anxiety, animal wellbeing, welfare assessment, non-invasive tool, animal management

## Abstract

Self-directed behaviors (SDBs), such as scratching, self-grooming, and body shaking, have been widely used as indicators of anxiety and social stress in non-human primates. However, research focused on SDB outside the primate range is still in infancy. Expanding this topic to other species can support animal welfare assessments and enhance comparative social studies. This study investigates whether SDB levels can reflect the social stress experienced in Asian elephants (*Elephas maximus*). Using all-occurrences and focal sampling on four captive individuals, we compared post-conflict SDB levels in victims to their baseline levels. Furthermore, changes in group composition during the study allowed us to examine whether individual baseline SDBs varied with social stress, measured as victimization rates across settings. Finally, we assessed whether there was any relation between levels of SDBs and stereotypic behavior. Results showed an increase in SDB levels in the victims after aggression compared to baselines, especially for behaviors recorded as counts. An estimated 39.8% increase in expected SDB counts was associated with the post-conflict context (E = 0.335, *p* = 0.024). The SDBs that differed more prominently were touch mouth, head shake, dust bathing, and trunk curled inwards (*p* < 0.05). Regarding baselines, two individuals increased their basal SDB levels when their rates as victims were the highest, although only one reached marginal significance. An individual who was never recorded as the recipient of aggression revealed remarkably low SDB levels. This study identified specific SDBs induced by social stress in Asian elephants and suggests SDB as a potential good indicator of their wellbeing.

## 1 Introduction

One of the main challenges in non-human animal research and animal welfare studies is to get information on the mental states of the individuals, particularly their negative internal experiences. Self-directed behavior (SDB) has been a promising non-invasive tool, largely used in non-human primates (1), which has not been fairly explored for a wide range of species; it is a form of auto-related displacement activity, often characterized as an abnormal pattern (e.g., a certain frequency, duration, or intensity) that appears to lack a clear function in the ongoing context (1). A reduction of displaced behaviors in macaques (*Macaca fascicularis*) by anxiolytic treatment was reported in some studies (2, 3) supporting the notion that there is a connection between anxiety-related states and displacement activities, namely SDB. Activities, including body care or “comfort displays” such as scratching, have been the most common SDBs assessed (4, 5). Studies on non-human primates suggest an increase in SDB levels in response to cognitively challenging tasks (6), disruption of temporal regularity (7), or predation risk (8). Furthermore, studies suggest that SDBs tend to increase after agonistic interactions in various non-human primate species (5, 9, 10). SDB was also connected to dominance rank and uncertainty of being the target of aggression (5, 11).

In social animals, specifically, social stress is an intrinsic aspect, encompassing the psychological and physiological mechanisms that emerge from interactions with the social environment, such as social threats or unbalanced relationships, which can profoundly influence an individual’s mental and physical health (12). Furthermore, social stress is transmissible, which can propagate and multiply negative mental states within a social unit (13). Therefore, behavioral tools sensitive to social stress can play a substantial role in the wellbeing of captive animals and can also provide valuable insights into sociality in free-ranging species.

Given the limited research on SDB in non-primate species, expanding its study could be valuable for improving welfare assessments and comparative research. Although still in its early stages, SDB in non-primates has already been observed in contexts of heightened stress or anxiety [e.g., horses (*Equus caballus*): (14); ravens (*Corvus corax*): (15); pigs (*Sus scrofa*): (16)]. Further studies on non-primate species will enhance our understanding of the link between SDB and the stress spectrum as well as its underlying mechanisms. Elephants are interesting subjects since studies report they are highly social (17) and possess advanced cognitive abilities (18, 19). Yet, there is remarkably scarce research on anxiety and mental states in elephants, especially on Asian species. Two studies investigated SDB levels in semi-captive African elephants (*Loxodonta africana*). They reported an increase in SDB rates as an outcome of amplified tourism (20), but no relation with physiological stress (i.e., cortisol levels) was found (21). Recently, a study focused on the personality of male African elephants reported that “self-directed anxious behavior” decreased in the presence of the dominant and young males (22), suggesting a modulation according to social dynamics. Overall, despite being stated that displacement (23) and comfort behaviors (24) are potentially valuable tools for the investigation of elephants’ wellbeing, scientific evidence on the connection between SDB and the internal experiences of Asian elephants is still limited [but see also (25)].

When discussing the internal experiences of non-human animals, physiological measures [e.g., cortisol levels (26)] and stereotypic behavior (SB) are commonly used tools (27, 28). Although, despite their shared association with the broad field of stress, SB and SDB may reflect distinct mental states and underlying mechanisms (3). Systematically assessing and comparing these behavioral categories can provide valuable insights and refine our understanding of SDB (29). SB, typically defined as repetitive, unvarying, and functionless (27), is frequently considered a pathological display (30). It can be induced by prolonged adverse circumstances and may persist even after the initial trigger is removed (31). Alternatively, SDBs are associated with acute stress or anxiety (1), making them highly relevant for identifying early signs and preventing escalation into more serious welfare concerns. From this perspective, it is important to consider SDB responses following a stressor and whether persistent stressors or adverse environments are sufficiently intense to influence baseline SDB levels.

This study investigates whether SDB can reflect the social stress experienced in Asian elephants. Specifically, two dimensions of SDB were examined: (1) SDB levels associated with a social stress event (aggression) and (2) basal SDB levels (baseline) in the absence of a clear distressing occurrence. For this, behavioral observations were conducted on four captive elephants. Throughout the study, specific individuals were removed from the group at different stages, creating three distinct group compositions. First, we examined whether SDB levels in victims increase following an agonistic interaction compared to baseline levels. Then, we explored the relationship between the aggressor-oriented trunk and the SDB performance in victims of aggression. Since social defeat is typically associated with increased avoidance and fear (32), we predicted that SDB values are lower when aggressor-oriented trunk levels are higher. Furthermore, we investigated whether SDB baselines vary according to the social stress experienced by each individual, as reflected by their victimization rates, in each group composition. This allowed us to examine whether heightened social stress could be detected in basal SDB levels, even without an immediate trigger/stressor. Finally, the relation between SDB and stereotypic behavior was also explored.

## 2 Methods

### 2.1 Study subjects and housing

The study was conducted on a group of four elephants at the Kyoto City Zoo, Japan. The main group is composed of four females, Harumi (HAR), Natsumi (NAT), Fuyumi (FUY), and Mito (MIT) (born in 2010, 2010, 2008, and 1971, respectively), and one male, Akito (born in 2011), separated by a fence. HAR, NAT, and FUY were born in Laos and brought together to Kyoto in 2014 to join MIT, which has been at the Kyoto City Zoo since 1979 (33). Only the females were considered for data collection and kept together in one fenced area in normal settings. Per normal zoo management procedures, FUY was removed from the main group for a female–male pairing in the adjacent fenced area for approximately 3 weeks in August 2022. Furthermore, MIT had a foot injury in October and was separated for about 6 weeks.

### 2.2 Data collection

Data was collected between August and November 2022 at Kyoto City Zoo, Japan. Non-invasive, direct behavioral observations on the elephant group were performed an average of three times a week, depending on the observer’s availability. It was not possible to carry out observations for some weeks. Observations on the whole group were supported by video and voice recordings using a Panasonic HC-W590MS camera. A total of 70 h were collected during 45 direct observations. Each was recorded in video format, with an average observation duration of 1 h 32 min (ranging from 14 min to 2 h 49 min). Observations were performed between 09:38 AM and 03:05 PM. The most frequent observation intervals were 10:00–10:59 and 11:00–11:59.

During observations, details of social conflicts (timings, combatants, contexts) and other relevant situations were noted and all 70 h of video recordings were later watched to document the required behaviors (SDB, SB and trunk oriented to aggressor) using Behavioral Observation Research Interactive Software (BORIS) [v 8.20.4 (34)].

#### 2.2.1 SDB, SB, and social attention after social conflicts

A social conflict was defined as the occurrence of an aggression (agonistic interaction) performed by the individual A (aggressor) toward the individual B (victim). The main aggressions recorded were kicking another individual with the leg, charging, usually followed by chasing, pulling another individual’s tail, biting, and pushing another individual with a raised head (35, 36).

Upon a social conflict, SDBs were collected using all-occurrences sampling on the victim for a period of 10 min (post-conflict block) following the ethogram (Table 1). The work of Manning et al. (20) was used as a reference to build the SDB ethogram, but some definitions were adapted to our species, and additional SDBs were tested. All behaviors were coded as events (i.e., counts) except trunk inside the mouth and trunk curled inwards, which were considered durations (i.e., state behaviors). If a renewed aggression occurred after 3 min of the initial aggression, it was considered an independent event, and both post-conflict blocks were considered. Consequently, five events had post-conflict blocks that lasted less than 10 min (i.e., 7, 6, 4, 5, and 6 min).

**Table 1:** Ethogram of the SDB collected on the study subjects.

Behaviors	Definition
Event behaviors	
Touch mouth	The trunk tip or the sides of the end of the trunk make contact with the lips or the corner of the mouth
Touch ear	The trunk tip or the sides of the end of the trunk make contact with the ear
Touch eye	The trunk tip or the sides of the end of the trunk make contact with the eye
Touch head	The trunk tip or the sides of the end of the trunk make contact with the head (includes the temporal glands)
Touch trunk	The trunk tip or the sides of the end of the trunk make contact with upper areas of the trunk
Touch leg	The trunk tip or the sides of the end of the trunk make contact with the leg (including foot)
Trunk swing	Trunk side to side or forward to back at least twice
Leg swing	The foot moves back and forth at least twice without hitting the ground
Dust bathing	The trunk throws dust or mud toward the own body (does not include water)
Leg lift	A movement with foot, lifting it and holding it for at least 2 s, without hitting the ground
Head shake	Rapidly shake or toss the head in a swift rotating motion
State behaviors	
Trunk inside the mouth	Trunk is in the mouth, between lips, without holding anything
Trunk in-curled	The trunk is rolled inwards at least 500 ms. The individual is not holding anything

It contains the name and description of the individual behaviors.

In addition, as a measure of social attention toward the aggressor, the duration of the victim’s trunk held in the direction of the aggressor was also recorded within the post-conflict block using all-occurrences sampling.

After a social conflict, SBs (Table 2) were also registered using all-occurrence sampling and extracted as durations (state behaviors).

**Table 2:** Ethogram of the stereotypic behaviors (SB) collected on the study subjects.

Behaviors	Definition
Swaying	Steady and continuous movement from side to side usually involves the head and part of the upper body
Head bobbing	Steady and continuous head movement from up to down

It contains the name and description of the individual behaviors.

Once most SDBs are trunk-related behaviors, when the focal’s trunk was not perfectly visible for detailed behavioral collection, even though all the other parts of the body were, the code ‘of’ was recorded. Its duration was later subtracted from the 10-min blocks to calculate the actual observation time during which the trunk was entirely in sight during post-conflict.

#### 2.2.2 Baselines for SDB and SB

The sampled periods were divided into three conditions according to the changes in the social settings at the Zoo. Condition 1 corresponds to the normal conditions where the elephant group is usually kept: four females together and the male separated. Condition 2 refers to the male–female pairing: three females together and a female (FUY) were held with the male. Condition 3 corresponds to the period when a female (MIT) was separated: three females together, one female (MIT) in another area, and one male in a different fenced area.

To assess basal levels, focal sampling blocks were performed without any distress to collect all occurrences of SDB and SB on each individual. Given the poor visibility in some videos for detailed behavioral sampling, the minimum duration of each sampling block was set to 2 min as long as each block fits the following requirements: at least 20 min after the start of the group observation; at least 20 min after last distress event (social conflict or disturbance); no collection during the feeding period in which the caretakers bring the food in and during the 30 min before the feeding period starts; sampling blocks were randomly selected; blocks should ideally be evenly distributed among the sampled days and during sampled periods (morning and afternoon). An attempt was made to reach 3 h of baseline per condition for each female (total 9 h/individual).

#### 2.2.3 Social stress faced by each individual

According to the removal of specific individuals in each setting, the social dynamics in the group changed, resulting in different targets of aggression in each condition. Thus, the rate at which each individual was a victim (received aggression from another individual) in each condition was calculated by dividing the number of times a certain elephant was the recipient of aggression by the total observation time in the corresponding condition.

### 2.3 Data analysis

Rstudio [v 2024.09.0 (37)] and R [v 4.2.2 (38)] were used to carry out all statistical analyses. To account for multiple comparisons and avoid multiple testing, each model was compared to a null model (39) with the same structure, including the same random effects and offsets as the original models (i.e., full models). Then, the likelihood ratio test (LRT) was applied using the R function “anova” with the argument “Chisq” to perform the null-full model comparisons. The original model was considered significant when the null-full comparisons yielded a *p*-value at the threshold of 0.05. The complete information on all models, including random effects, is reported in the Supplementary material. The fit of all models was checked using the diagnostic tool DHARMa (DHARMa package).

#### 2.3.1 SDB levels following a social conflict

##### 2.3.1.1 Investigating whether SDB levels increase after receiving an aggression

A Generalized Linear Mixed Model (GLMM) with a Poisson distribution (glmmTMB package) was applied to analyze SDB levels in recipients of aggression. Counts of each SDB were the response variable, and the number of minutes of the observation block was added as an offset term. The context (post-conflict or baseline) was set as the fixed effect. The behaviors of SDB and ID were included as random effects. The individual levels of each SDB assessed in the post-conflict blocks were matched with the individual baseline from the corresponding condition in which the conflicts happened. As the model showed overdispersion, it was run using a Negative Binomial distribution (glmmTMB package).

For SDBs collected with durations (state behaviors), a Generalized Linear Mixed Model (GLMM) with a Beta distribution (glmmTMB package) was applied. Proportions of SDBs’ durations (duration of behavior/total duration of observation) were the response variable, and the context (post-conflict or baseline) was set as a fixed effect. Behavior and ID were included as random effects. Again, the SDB levels from each conflict were matched with the baseline from the corresponding condition in which the conflict happened.

To test for the differences between baselines and post-conflict periods on each behavior, the variable behavior was added as an interaction term with the context on the previous models, and estimated marginal means analysis (emmeans package) was applied as a *post-hoc* analysis. The *p*-values were adjusted using the Tukey method.

##### 2.3.1.2 Relation between SDB and aggressor-oriented trunk after social conflicts

The counts of all SDBs per individual after each conflict were pooled together. The total rates for each post-conflict event were calculated using the formula counts/total observation time. Time proportions of self-directed state behaviors and trunk toward the aggressor were assessed using the formula behavior durations/total observation period in the post-conflict blocks. To assess the relation between SDB rates and the trunk toward aggressor in victims during the post-conflict periods, two Spearman correlations were applied to rates of SDB involving only counts and proportions of SDB including durations.

##### 2.3.1.3 Relation between SDB and SB after social conflicts

The proportion of time of stereotypic behaviors in the victims after the social conflicts was calculated by dividing the durations of stereotypic behaviors by the total observation time in the post-conflict blocks. To investigate a potential relationship between rates of SDB and stereotypic behavior, two Spearman correlations were applied, separately, one to SDB involving only counts/min and another to proportions of SDBs.

#### 2.3.2 SDB baselines among conditions

##### 2.3.2.1 Differences in the individual baselines between conditions

The differences between the SDB baselines from each condition were investigated for each individual. Only event SDBs (i.e., counts) were used in this section, given the small dataset of state SDBs (i.e., durations).

A Generalized Linear Model (GLM) with a Poisson distribution (lme4 package) was run with the total number of SDBs recorded on each sampling block as the response variable, the interaction term between ID and condition as the fixed effect and the number of minutes of the observation of each block was added as an offset term. As the model showed dispersion, it was run using a Negative Binomial distribution (MASS package). *Post-hoc* pairwise comparisons were conducted using estimated marginal means (emmeans package) to examine differences in the SDB baselines between conditions for each individual. The results were back-transformed from the log scale and presented in the original scale for interpretability. The *p*-values were adjusted using the Tukey method.

Differences in the levels of SB between conditions were also assessed so that each individual could have a general overview of the fluctuation of SDB and SB throughout the study period. Given that stereotypic behaviors were collected as durations, data were highly skewed and zero-inflated, a Generalized Linear Model (GLM) with a Tweedie distribution (glmmTMB package) was deemed appropriate. The duration of stereotypic behaviors was included in the model as the response variable, while the interaction term between condition and ID was the predictor. The observation time was included as an offset term. Similarly to the previous model, *post-hoc* pairwise comparisons on estimated marginal means (emmeans package) were calculated to examine differences in the baselines of the stereotypic behaviors between conditions for each individual. The results were back-transformed from the log scale to the original scale, and *p*-values were adjusted using the Tukey method.

##### 2.3.2.2 Relation between SDB and SB baselines

To investigate any connection between SDB levels and SB, a Spearman correlation was applied to the rates of SDB (counts/min) and the proportion of time of SB from each focal sampling block.

## 3 Results

A total of 70 h of observation time (condition 1: 30 h; condition 2: 20 h; condition 3: 20 h) and 32 instances of social conflict were recorded.

To construct individual SDB baselines, a total of 301 focal sampling blocks, ranging from 2 to 34 min, were collected. For conditions 1 and 2, each individual had a baseline of 3 h per condition. Due to visibility issues in condition 3, HAR recorded 2 h and 28 min, NAT 2 h and 22 min, and FUY 2 h and 50 min. A baseline for MIT was not calculated for condition 3 due to her confinement within the enclosure for recovery from injury.

### 3.1 SDB levels after a social conflict

#### 3.1.1 Does SDB increase after an individual receives aggression?

**Figure 1:**
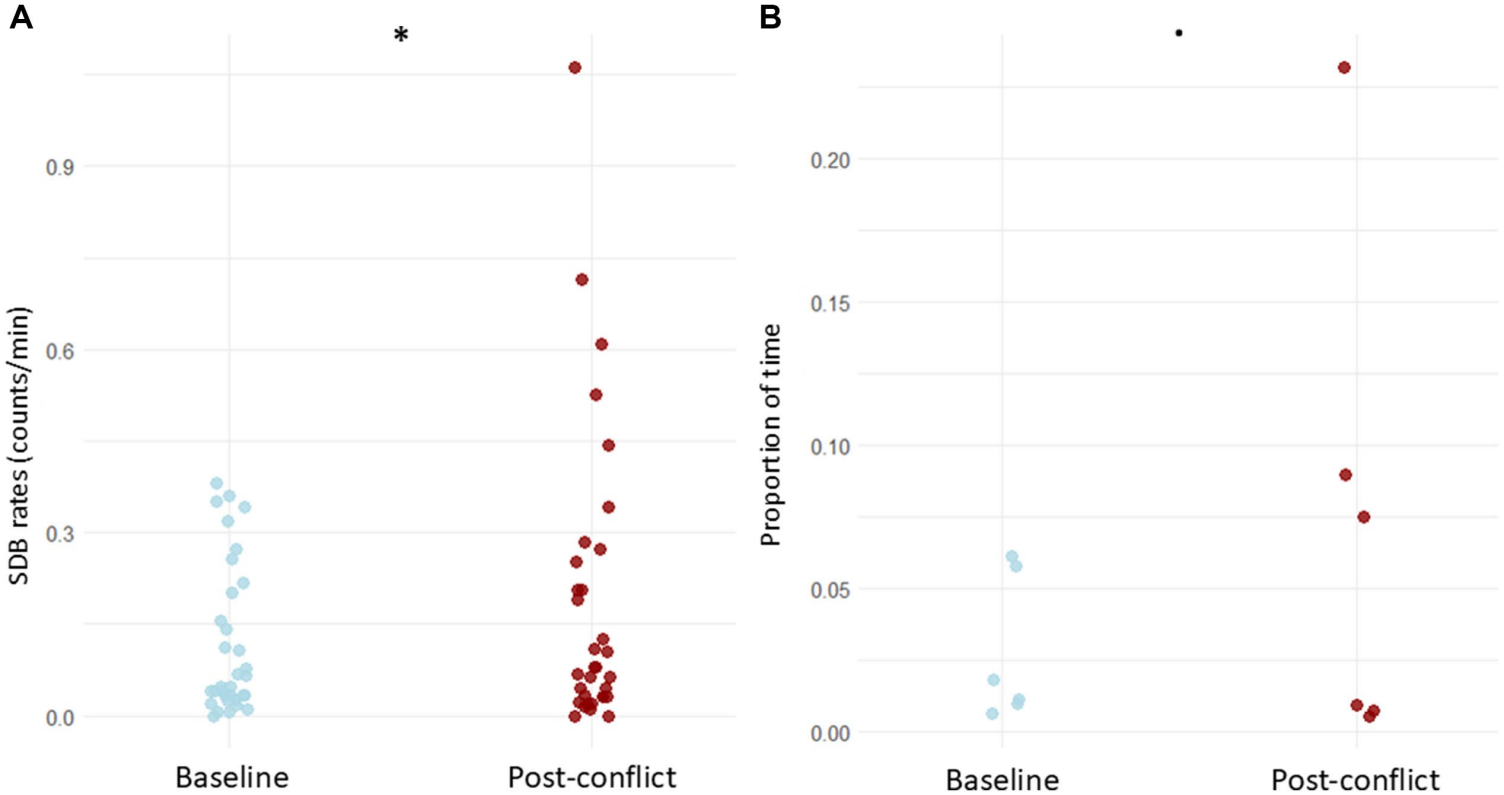
SDB levels in the victims among baseline and post-conflict contexts. **(A)** Comparison of event SDB levels (counts/min). **(B)** Comparison of state SDB levels (proportions of time). Datapoints represent the levels of each SDB assessed per each individual. *p < 0.05; •*p* < 0.10.

**Figure 2:**
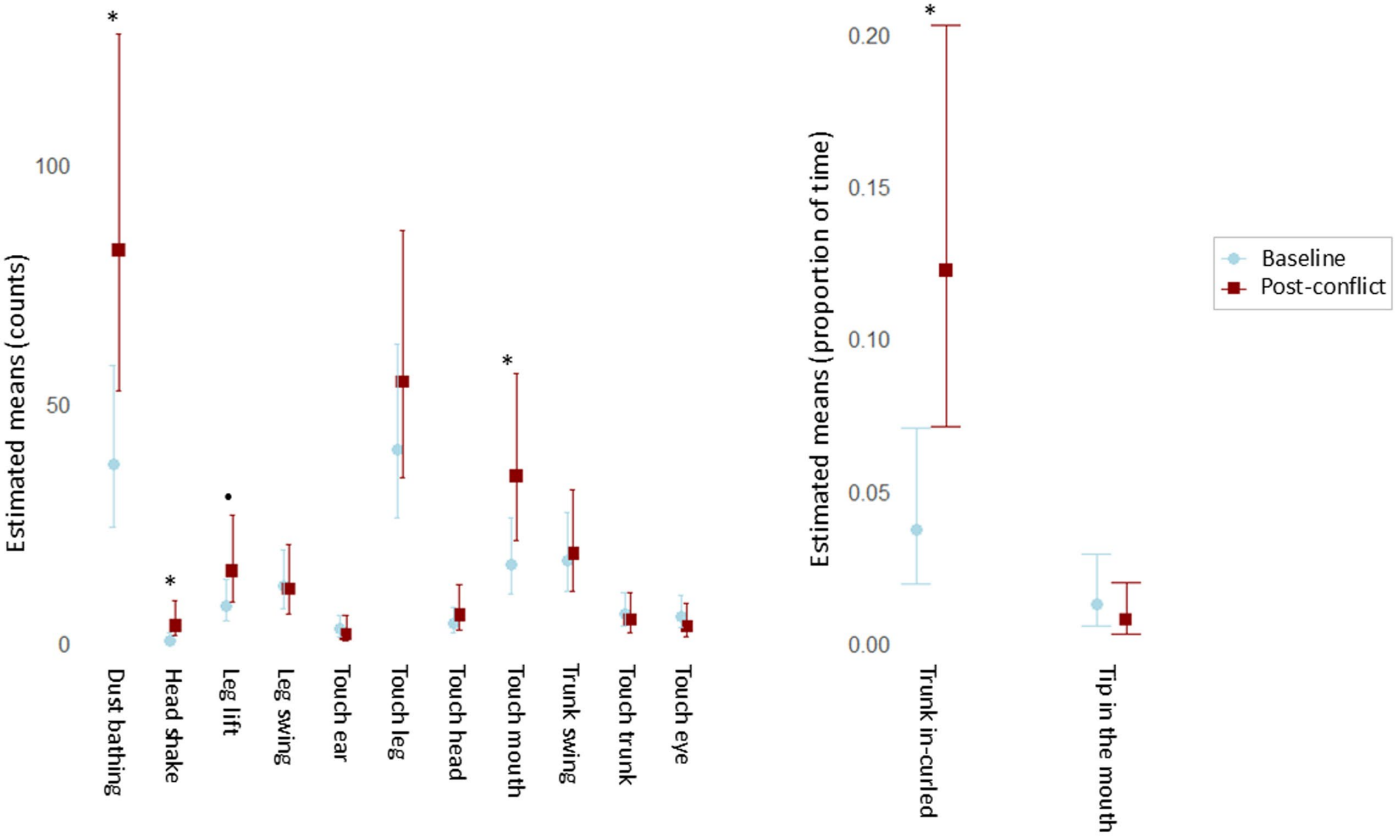
Pairwise comparisons to investigate the influence of the context (baseline vs. post-conflict) on the levels of each SDB. **(A)** Event SDBs (estimated counts) with 95% confidence intervals. **(B)** State SDBs (estimated proportions of time) with 95% confidence intervals. *p < 0.05; •*p* < 0.10.

The LRT applied to compare the null and full models revealed that the context (baseline vs. post-conflict) had a significant influence on the SDB counts (x^2^ = 145.944, df = 3, *p* < 0.001). The original GLMM showed significantly higher counts of SDB during post-conflict (Figure 1A) than during the baselines (E = 0.335, *p* = 0.024; Supplementary Table S1), corresponding to an estimated 39.8% increase in expected SDB counts. The follow-up GLMM, including the interaction between context and behavior as a predictor, was also significant according to the LRT result (x^2^ = 160.625, df = 20, *p* < 0.001). The estimated pairwise comparisons showed that dust bathing (ratio = 0.456, *p* = 0.013), head shake (ratio = 0.132, *p* = 0.020) and touch mouth (ratio = 0.469, *p* = 0.028) increased significantly after the victim received an aggression when compared to basal levels (Figure 2A). In addition, leg lift showed a prominent difference; though not significant at *p* < 0.05, it was significant at *p* < 0.10 (ratio = 0.510, *p* = 0.098; Supplementary Table S2.1).

Regarding state SDBs, LRT on the null full models’ comparison revealed a marginal significance of the context (x^2^ = 3.628, df = 1, *p* = 0.057), most likely due to the small number of assessed behaviors. Nonetheless, the GLMM (Supplementary Table S3) showed higher durations of SDB after conflicts than in the baselines (Figure 1B). Furthermore, in the follow-up model in which the behavior was included as an interaction term with the context, the LRT on the null full models’ comparison revealed high significance (x^2^ = 20.064, df = 3, *p* < 0.001). This suggests that each SDB pattern highly influences the overall SDB levels between contexts. The pairwise comparisons demonstrate that the duration of the behavior trunk curled inwards significantly increased in the post-conflict period (ratio = 0.277, *p* < 0.001) (Figure 2B).

#### 3.1.2 SDB and trunk and aggressor-oriented trunk after social conflicts

**Figure 3:**
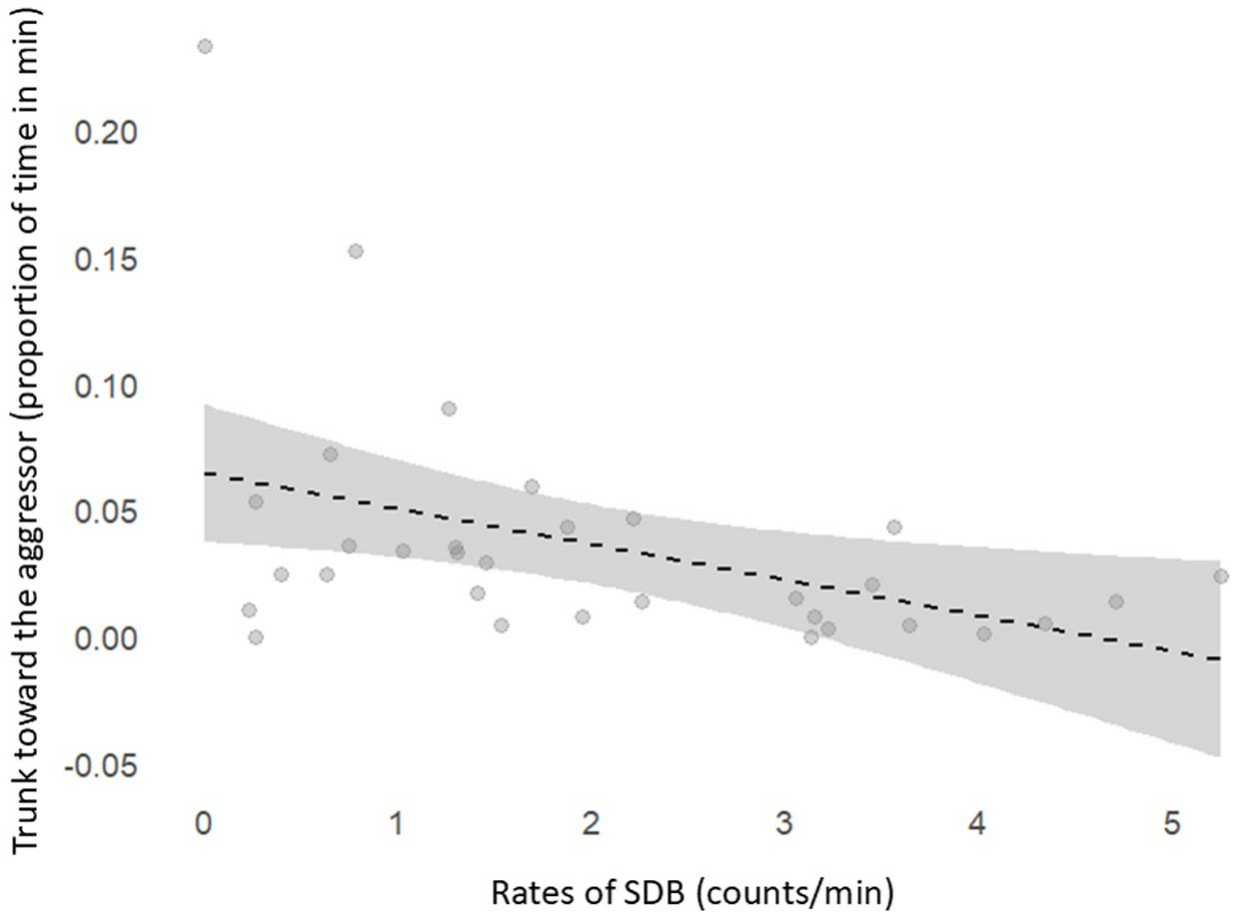
Negative correlation between SDB rates (counts/total observation time) and proportion of time the trunk was held toward the aggressor (duration of trunk oriented to aggressor/total observation time) after social conflicts.

Results revealed a negative correlation between event SDB rates and the levels of trunk toward aggressor (r = −0.45, *p* = 0.01) in the victims during post-conflict blocks (Figure 3). However, no relation was found between state SDBs and trunk toward aggressor (r = 0.03, *p* = 0.86).

### 3.2 Baselines among conditions

#### 3.2.1 Social stress across conditions

According to the variations in the social settings, the dynamics of aggression also changed. MIT was the aggressor in condition 1 (16 aggressions out of a total of 17 aggressions) and in condition 2 (3 aggressions out of 3). During condition 3, in the absence of MIT due to injury, the main aggressor became FUY (11 aggression in a total of 12). The main targets of agonistic interactions also differed among conditions (Table 3). In normal settings (condition 1), FUY is the main victim. However, during condition 2, she was separated from the main group, and the overall occurrence of social conflicts decreased. In condition 3, the agonistic interactions increased again, and the main recipient of aggression became HAR (Table 4).

**Table 3:** Rates (events per hour) for each individual as a victim of aggression and the total rate of occurrence of social conflicts per condition.

ID	Condition 1	Condition 2	Condition 3	Mean rate	Variance	SD
MIT	0.00	0.00	na	0.00	0.00	0.00
HAR	0.00	0.00	0.55	0.18	0.10	0.32
FUY	0.40	0.00	0.00	0.13	0.05	0.23
NAT	0.17	0.15	0.05	0.12	<0.01	0.06
Total	0.57	0.15	0.60	–	–	–

**Table 4:** Overall data for individual baselines: total number of attacks toward each individual; total rates of SDB in counts per minute and total proportion of minutes (total time performing SB/total observation time) each elephant spent performing SB in each condition.

ID	No. of attacks experienced			Total rates of SDB (counts/min)			Total time proportion of SB (minutes)		
	Condition 1	Condition 2	Condition 3	Condition 1	Condition 2	Condition 3	Condition 1	Condition 2	Condition 3
MIT	0.00	0.00	na	0.46	0.59	na	0.20	0.02	na
HAR	0.00	0.00	11.00	1.20	1.01	1.33	0.00	0.00	0.00
FUY	12.00	0.00	0.00	1.45	1.01	0.90	0.50	<0.01	<0.01
NAT	5.00	3.00	1.00	1.44	0.64	1.28	0.00	0.00	0.00

#### 3.2.2 Differences in the individual baselines between conditions

**Figure 4:**
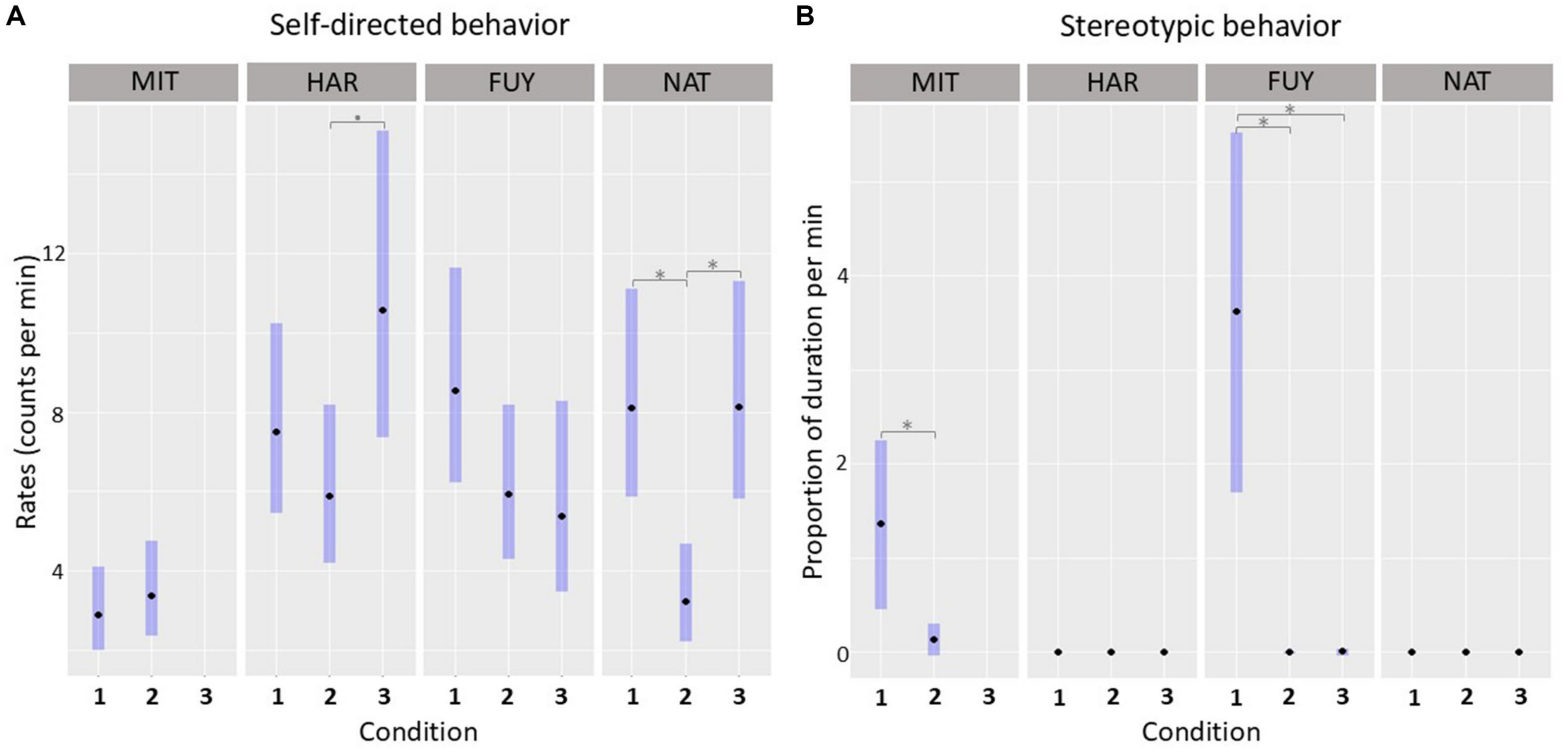
Patterns of SDB and SB throughout the study period. **(A)** Comparison of the SDB levels of each individual between conditions. **(B)** Comparison of the stereotypy levels of each individual between conditions. Estimated means are expressed with 95% confidence intervals. *p < 0.05; •*p* < 0.10.

The likelihood ratio test (LRT) on the null and full GLM comparison revealed that the interaction term between ID and condition had a significant influence on the SDB levels (x^2^ = 55.047, df = 10, *p* < 0.001; complete model outcome on Supplementary Table S5). The contrasts in the *post-hoc* analysis (Figure 4A) showed that the FUY’s SDB baselines did not differ among conditions: cond1-cond2 (ratio = 1.435, *p* = 0.257); cond1-cond3 (ratio = 1.583, *p* = 0.210); cond2-cond3 (ratio = 1.103, *p* = 0.932). SDB levels of MIT also did not differ between cond1 and cond2 (ratio = 0.857, *p* = 0.541). HAR’s SDB baseline from cond3 differed from cond2 (ratio = − 0.558, *p* = 0.051), but, curiously, not from cond1 (ratio = 0.710, *p* = 0.336). Her levels did not significantly differ between cond1 and cond2 (ratio = 1.273, *p* = 0.553). Finally, NAT’s SDB levels in cond2 differed significantly from cond1 (ratio = 2.502, *p <* 0.001) and from cond3 (ratio = 0.398, *p <* 0.001), but estimated means in cond1 and cond3 did not change significantly (ratio = 0.996, *p* = 0.999).

Regarding stereotypic behavior, the LRT on the null and full GLM comparison also revealed that the original model containing the interaction term between ID and condition was significant (x^2^ = 118.796, df = 10, *p* < 0.001; complete information in Supplementary Table S6). The contrasts in the *post-hoc* analysis (Figure 4B) showed that only two individuals, FUY and MIT, displayed stereotypic behavior throughout the study period. FUY showed significant differences among condition 1 and condition 2 (ratio = 931.788, *p <* 0.001), and between condition 1 and condition 3 (ratio = 337.452, *p <* 0.001), but not between condition 2 and condition 3 (E = 0.362, *p* = 0.885). The stereotypic behavior levels of MIT also differed significantly between the two conditions assessed (cond1-cond2: ratio = 9.619, *p* = 0.001).

### 3.3 Relation between SDB and SB

The Spearman test showed no correlation between levels of stereotypic behavior (proportion of time) and event SDBs (r = 0.06, *p* = 0.75) or state SDBs (r = 0.03, *p* = 0.87) after social conflicts. This suggests that there was no apparent relation between the display of SDBs and SB by the victims after receiving aggression.

Regarding baseline levels, there was also no correlation between rates of event SDBs and proportion of time of SB in the baseline blocks (r = 0.06; *p* = 0.34) of the four individuals.

## 4 Discussion

In this study, the influence of social stress on SDB levels was evaluated on two dimensions, in post-conflict and the absence of any clear stressor, among distinct group compositions. The results showed an overall increase in the SDB levels after the individuals had received an aggression compared to their SDB basal levels. This was more evident for SDBs collected as counts than for SDBs recorded as durations (Figure 1). Moreover, dust bathing, head shake, touch mouth, and trunk in-curled were particularly sensitive to the context of social stress (Figure 2). These results suggest a connection between SDBs and the distress or anxiety experienced by the victims after social conflicts. Furthermore, the identity of the individuals did not influence the observed pattern (Supplementary Tables S1, S3). Self-directed behaviors may be, therefore, a good behavioral tool to measure the emotional experience associated to post-conflict in Asian elephants which aligns with studies carried in other species [e.g. long-tailed macaques (*Macaca fascicularis*): (40); brown capuchin monkeys (*Cebus apella*): (10); Japanese macaques (*Macaca fuscata*): (41)]. The uncertainty of a renewed agonistic interaction (40) and the consequences of the relationship disruption (41) have been hypothesized as the two main causes of post-conflict anxiety leading to elevated SDB on recipients of aggression. On the other side, the physiological and behavioral effects related to the activity of the autonomic nervous system of a stress response induced to react to a stressor (i.e., aggression) could also account, to some extent, for elevated SDB levels in this context (9).

The temporary removal of individuals from the main group provided an opportunity to examine fluctuations in SDB baselines in response to different social dynamics, offering insights into individual experience and perception. The baseline patterns observed were consistent with a connection between SDBs and individual social stress, where higher levels generally corresponded to elevated SDB values. However, statistical significance was not always reached. As expected, the individual MIT exhibited consistently low SDB basal levels throughout the study, as she never received an aggression (Figure 4A). Regarding HAR, her highest SDB basal levels were recorded during condition 3, when she experienced the highest rate of aggressions (Table 3; Figure 4A), further corroborating the link between SDBs and social stress. The SDB rates were marginally significant from condition 2, but not from condition 1 (Figure 4A). Since the baselines reflect the SDB levels without any apparent stressor, they likely reflect the anxiety experienced associated with the increased uncertainty about aggression. If an individual is subject to a higher number of aggressions within a certain period of time, the likelihood of receiving an imminent aggression at any given moment becomes unclear. This increased uncertainty about the future can be interpreted as anxiety and reflected in heightened SDB levels (42). Similarly, FUY exhibited the highest SDB levels during condition 1, when she had the highest victimization rate (Table 3; Figure 4A). However, differences in her baseline SDB across conditions did not reach statistical significance. When interpreting this result, it is important to consider that condition 1 represents the group’s typical social settings for most of the year. Given that FUY is repeatedly the main target of aggression, her exposure to social stress may have become chronically persistent. Prolonged exposure to the same stressors is known to disrupt and alter the normal functioning of physiological and behavioral mechanisms connected to stress and anxiety (43), which could influence SDB expression across conditions. Furthermore, the cumulative effects of social stress may, at least, help explain why FUY (15 years old) developed stereotypic behavior. Moreover, although FUY did not experience any aggression during condition 2, she was unusually placed in a female–male pairing, which can itself represent a different form of social stress. This could have also influenced the SDB levels for this individual, potentially obscuring significant differences between conditions.

Interestingly, NAT, who had relatively low and evenly distributed rates as a victim throughout the study (Table 3), showed significant differences in SDB baselines between conditions. Her higher SDB levels were recorded during conditions 1 and 3 (Figure 4A) when the occurrence of social conflict was more frequent (0.57 event/h and 0.60 event/h, respectively) compared to condition 2 (0.15 event/h). This suggests that NAT could have been more sensitive to the group’s overall social stress. One potential explanation for this result relies on the assumption that NAT may be a more neutral individual in the group and its social dynamics. She did receive occasional aggressions throughout the study period, but she was never intensively targeted. Therefore, she may not have experienced severe anxiety regarding the imminence of an agonistic interaction toward herself. Given that the group is small and all the individuals live closely, more neutral individuals, such as NAT, who were never an aggressor or the main target of aggression, may be impacted by others’ conflicts, disrupting the dynamics and harmony in the captive group. This interpretation is aligned with studies reporting that witnessing aggression increased SDB rates of bystanders in non-human primates (44, 45). Such effects may be facilitated by mechanisms related to emotional contagion and/or concern for others (46, 47). While these phenomena have not been extensively investigated in elephants, a study on captive Asian elephants found that they reassure conspecifics in distress (48), suggesting sensitivity to other’s emotional states. Furthermore, Bates et al. (49) discussed various observational reports and proposed that elephants possess high empathic abilities.

Nonetheless, other factors could have influenced these results, such as relationships and affiliative social interactions (50). In addition, the absence of significant differences among conditions for some individuals could indicate that their exposure to social stress was not strong or long enough.

In this study, only two individuals showed SB. Contrary to FUY, whose SB levels significantly decreased as the victimization rate decreased, the display of SB by MIT did not appear to be linked to receiving aggression, as she was never recorded as a victim (Table 3). The causes that led to the development of SB are most likely different for FUY and MIT (33). However, it is very difficult to make any clear inferences according to the circumstances provided. Furthermore, no direct relationship between SDB and SB was found, either after social conflicts or in the baselines. This result does not support the existence of a relation between the performance of SDB and SB. Instead, they are aligned with the idea that these behaviors may reflect distinct welfare aspects - SB might represent a cumulative state while SDB a more immediate state (4). However, SB combined with other measures may still be useful for captive welfare. While SDB and stereotypic displays appear to be linked to the stress spectrum, they may signify different aspects.

Additionally, results revealed a negative correlation between SDB rates and the duration of victims holding their trunks toward aggressors after social conflicts (Figure 3). This indicates that SDB values were lower when the trunk orientation toward the aggressor was more prolonged. Following an aggression, the heightened anxiety about the possibility of renewed aggression can lead to an increased attentional focus on the aggressor’s behavior. Literature states that after social defeat, victims tend to increase vigilance and fear (32). Further studies report an increased attentional bias toward the emotional stimuli of conspecifics to prepare for a threat (51), and Cooper et al. (52) reported that victims avoided aggressors after social conflicts. Due to their highly developed olfaction and chemical sensing systems, elephants’ trunks are very important for gathering information about their surroundings (53), including conspecific behavior (54). Therefore, since most SDBs are also trunk-related behaviors, increased trunk use for attentional bias toward an aggressor may decrease SDB rates, as suggested in our results. This could work as a potential confounder when assessing SDB as a behavioral proxy in elephants for anxiety in specific contexts, which is unlikely to occur in primates once they rely primarily on visual cues for attentional states (51). These considerations underscore the importance of accounting for interspecific differences to develop ecologically valid measures. Adapting well-validated tools from primate-centric research may require some prudence. As most SDBs were trunk-related, it seems relevant to continue examining SDBs involving legs and head, as well as other potential trunkless behaviors.

Finally, we acknowledge that the small sample and zoo setting are limitations of our study, warranting caution when drawing strong conclusions. Based on our findings, we encourage further studies on SDB patterns in elephants, using larger sample sizes and incorporating additional factors such as social interactions and diverse contexts to deepen our knowledge and the reliability of the behavioral proxies.

## 5 Conclusion

This study suggested a link between SDB performance and the anxiety triggered by social stress in Asian elephants. Certain SDBs were significantly more induced in post-conflict contexts. This is the first systematic investigation of the connection between SDB and social stress in this species, contributing to a broader understanding of SDB beyond the non-human primates. Moreover, the results highlight the potential utility of SDBs in assessing individual internal state, with significant implications for welfare studies and comparative research.

## Data Availability

The original contributions presented in the study are included in the article/Supplementary material, further inquiries can be directed to the corresponding author.
