# Supplementary material for: Self-directed behavior reflects social stress in captive Asian elephants
Source: Front Vet Sci. 2025 Jun 13;12:1629664. doi: 10.3389/fvets.2025.1629664 (PMC12202216; doi:10.3389/fvets.2025.1629664)
Supplement: Supplementary file 1 [file Table_1.docx]

***Supplementary Material***

**Supplementary Data**

**Dataset S1.** Total counts of each event SDB assessed during all post-conflict blocks (pc) and baseline (bs) blocks. Dataset also contains the identity of the individuals and the total observation time (all blocks) for each individual during baselines and post-conflict blocks.

**Dataset S2.** Total duration of each state SDB assessed during all post-conflict blocks (pc) and baseline blocks (bs). Dataset also contains the identity of the individuals and the total observation time (all blocks) for each individual during baselines and post-conflict blocks.

**Dataset S3.** Rates of event SDB (counts/min), proportion of time of state SDB, proportion of time of stereotypic behavior (SB) and proportion of time of trunk held towards the aggressor after each conflict (during each post-conflict block).

**Dataset S4.** Counts of all SDB and durations of all SB per each block sampled for each individual baseline per each condition. Dataset also includes the rates of SDB (counts/min) and proportion of time of SB.

**Supplementary Tables**

**Table S1.** Outcome of the GLMM using a Negative Binomial distribution. **a)** Context [baseline (BS) vs post-conflict (PC)] was set as a fixed effect and SDB counts as the response variable. **b)** ID and behavior were included as random effects.

| **a)** |  |  |  |  |
| --- | --- | --- | --- | --- |
| **Terms** | **Estimate** | **SE** | **Z value** | **p value** |
| Intercept | -2.604 | 0.339 | -7.693 | >0.001 |
| PC | 0.335 | 0.149 | 2.253 | 0.024 |

| **b)** |  |  |
| --- | --- | --- |
| **Terms (random effects)** | **Variance** | **Std.Dev.** |
| ID | >0.001 | >0.001 |
| behavior | 1.142 | 1.068 |

**Table S2.** Outcome of the GLMM using a Negative Binomial distribution, with the interaction between context (baseline vs post-conflict) and behavior as a fixed effect and the counts of SDB as the response variable.

| **Term** | **Estimate** | **SE** | **Z value** | **p value** |
| --- | --- | --- | --- | --- |
| Intercept | -1.126 | 0.223 | -5.050 | >0.001 |
| PC | 0.784 | 0.317 | 2.478 | 0.013 |
| Head shake | -4.383 | 0.770 | -5.690 | >0.001 |
| Leg lift | -1.574 | 0.353 | -4.460 | >0.001 |
| Leg swing | -1.144 | 0.337 | -3.395 | >0.001 |
| Touch ear | -2.580 | 0.420 | -6.138 | >0.001 |
| Touch leg | 0.077 | 0.315 | 0.245 | 0.806 |
| Touch head | -2.251 | 0.390 | -5.772 | >0.001 |
| Touch mouth | -0.829 | 0.328 | -2.530 | 0.011 |
| Trunk swing | -0.781 | 0.326 | -2.394 | 0.016 |
| Touch trunk | -1.835 | 0.363 | -5.057 | >0.001 |
| Touch eye | -1.910 | 0.367 | -5.200 | >0.001 |
| PC: Head shake | 1.241 | 0.925 | 1.342 | 0.180 |
| PC: Leg lift | -0.123 | 0.509 | -0.242 | 0.809 |
| PC: Leg swing | -0.847 | 0.511 | -1.656 | 0.098 |
| PC: Touch ear | -1.290 | 0.779 | -1.653 | 0.098 |
| PC: Touch leg | -0.486 | 0.452 | -1.075 | 0.282 |
| PC: Touch head | -0.403 | 0.590 | -0.684 | 0.494 |
| PC: Touch mouth | -0.028 | 0.467 | -0.059 | 0.952 |
| PC: Trunk swing | -0.703 | 0.482 | -1.456 | 0.145 |
| PC: Touch trunk | -1.020 | 0.594 | -1.717 | 0.086 |
| PC: Touch eye | -1.277 | 0.630 | -2.025 | 0.043 |

**Table S2.1.** Outcome of the pairwise comparisons of estimated marginal means for each event SDB between post-conflict (PC) and baseline (BS), including contrast estimates (BS/PC). Tukey’s adjustment was used for multiple comparisons.

|  | **Estimated marginal means** | | | | | **Contrasts** | | | |
| --- | --- | --- | --- | --- | --- | --- | --- | --- | --- |
| **SDB** | **Context** | **response** | **SE** | **asymp.LCL** | **asymp.UCL** | **ratio** | **SE** | **Z** | **p value** |
| Dust bathing | BS | 37.466 | 8.350 | 24.206 | 57.990 | 0.456 | 0.144 | -2.478 | 0.013 |
|  | PC | 82.083 | 18.400 | 52.840 | 127.51 |  |  |  |  |
| Head shake | BS | 0.468 | 0.345 | 0.110 | 1.980 | 0.132 | 0.115 | -2.330 | 0.020 |
|  | PC | 3.546 | 1.630 | 1.438 | 8.740 |  |  |  |  |
| Leg lift | BS | 7.766 | 2.120 | 4.543 | 13.280 | 0.516 | 0.206 | -1.657 | 0.098 |
|  | PC | 15.044 | 4.370 | 8.509 | 26.600 |  |  |  |  |
| Leg swing | BS | 11.929 | 3.020 | 7.267 | 19.580 | 1.065 | 0.428 | 0.156 | 0.876 |
|  | PC | 11.205 | 3.500 | 6.076 | 20.66 |  |  |  |  |
| Touch ear | BS | 2.840 | 1.010 | 1.413 | 5.710 | 1.654 | 1.180 | 0.707 | 0.480 |
|  | PC | 1.717 | 1.060 | 0.514 | 5.740 |  |  |  |  |
| Touch leg | BS | 40.469 | 8.980 | 26.196 | 62.520 | 0.742 | 0.240 | -0.924 | 0.356 |
|  | PC | 54.531 | 12.800 | 34.440 | 86.340 |  |  |  |  |
| Touch head | BS | 3.945 | 1.260 | 2.107 | 7.390 | 0.683 | 0.340 | -0.766 | 0.444 |
|  | PC | 5.776 | 2.200 | 2.737 | 12.190 |  |  |  |  |
| Touch mouth | BS | 16.358 | 3.930 | 10.221 | 26.180 | 0.469 | 0.161 | -2.201 | 0.028 |
|  | PC | 34.864 | 8.580 | 21.522 | 56.480 |  |  |  |  |
| Trunk swing | BS | 17.165 | 4.080 | 10.766 | 27.370 | 0.922 | 0.336 | -0.224 | 0.823 |
|  | PC | 18.624 | 5.140 | 10.847 | 31.980 |  |  |  |  |
| Touch trunk | BS | 5.979 | 1.710 | 3.411 | 10.480 | 1.266 | 0.636 | 0.469 | 0.639 |
|  | PC | 4.724 | 1.950 | 2.103 | 10.610 |  |  |  |  |
| Touch eye | BS | 5.549 | 1.620 | 3.131 | 9.830 | 1.637 | 0.892 | 0.903 | 0.366 |
|  | PC | 3.391 | 1.560 | 1.375 | 8.360 |  |  |  |  |

**Table S3.** Outcome of the GLMM using a Beta distribution. a) Context [baseline (BS) vs post-conflict (PC)] as a fixed term and proportions of SDBs as the response variable. b) ID and behavior were included as random effects.

| **a)** |  |  |  |  |
| --- | --- | --- | --- | --- |
| **Terms** | **Estimate** | **SE** | **Z value** | **p value** |
| Intercept | -3.958 | 0.878 | -4.507 | <0.001 |
| PC | 0.892 | 0.338 | 2.643 | 0.008 |

| **b)** |  |  |
| --- | --- | --- |
| **Terms (random effects)** | **Variance** | **Std.Dev.** |
| ID | 0.216 | 0.465 |
| behavior | 1.072 | 1.035 |

**Table S4.** Outcome of the GLMM using a Beta distribution, with the interaction between context (baseline vs post-conflict) and behavior as a fixed and proportions of SDBs as the response variable.

| **Term** | | **Estimate** | **SE** | **Z** | **p value** |
| --- | --- | --- | --- | --- | --- |
| Intercept | | -3.255 | 0.347 | -9.395 | < 0.001 |
| PC | | 1.284 | 0.230 | 5.587 | < 0.001 |
| Trunk inside mouth | | -1.093 | 0.366 | -2.984 | 0.003 |
| PC: Trunk inside mouth | | -1.831 | 0.543 | -3.372 | 0.001 |
|  |  | |  |  |  |

**Table S4.1.** Outcome of the pairwise comparisons of estimated marginal means for each state SDB between post-conflict (PC) and baseline (BS), including contrast estimates (BS/PC). Tukey’s adjustment was used for multiple comparisons.

|  | **Estimated marginal means** | | | | | | **Contrasts** | | | |
| --- | --- | --- | --- | --- | --- | --- | --- | --- | --- | --- |
| **SDB** | | **Context** | **response** | **SE** | **asymp.LCL** | **asymp.UCL** | **ratio** | **SE** | **Z** | **p value** |
| Trunk in-curled | | BS | 0.037 | 0.012 | 0.019 | 0.071 | 0.277 | 0.064 | -5.587 | <0.001 |
|  |  | PC | 0.122 | 0.033 | 0.071 | 0.203 |  |  |  |  |
| Trunk inside mouth | | BS | 0.012 | 0.005 | 0.006 | 0.029 | 1.729 | 0.845 | 1.121 | 0.263 |
|  |  | PC | 0.007 | 0.004 | 0.003 | 0.020 |  |  |  |  |

**Table S5.** Outcome of the GLM using a Negative Binomial distribution with the interaction term between ID and Condition as a predictor. The rates of SDBs were the response variable and the observation time was included as an offset.

|  | **Estimate** | **SE** | **Z value** | **p value** |
| --- | --- | --- | --- | --- |
| Intercept | 0.281 | 0.160 | 1.757 | 0.079 |
| HAR | -0.129 | 0.226 | -0.573 | 0.566 |
| MIT | -1.083 | 0.242 | -4.474 | <0.001 |
| NAT | -0.053 | 0.228 | -0.231 | 0.817 |
| cond2 | -0.361 | 0.230 | -1.574 | 0.115 |
| cond3 | -0.460 | 0.272 | -1.687 | 0.0916 |
| HAR:cond2 | 0.120 | 0.327 | 0.368 | 7.131 |
| MIT:cond2 | 0.516 | 0.342 | 1.510 | 0.131 |
| NAT:cond2 | -0.556 | 0.339 | -1.639 | 0.101 |
| HAR:cond3 | 0.802 | 0.365 | 2.197 | 0.028 |
| NAT:cond3 | 0.464 | 0.359 | 1.292 | 0.196 |

**Table S5.1.** Outcome of the pairwise comparisons of estimated marginal means for the SDB rates of each individual between Conditions, including contrast estimates. Tukey’s adjustment was used for multiple comparisons.

| **Estimated marginal means** | | | | | | **Contrasts** | | | | |
| --- | --- | --- | --- | --- | --- | --- | --- | --- | --- | --- |
| **ID** | **condition** | **response** | **SE** | **LCL** | **UCL** | **contrast** | **ratio** | **SE** | **Z** | **p value** |
| MIT | 1 | 2.890 | 0.526 | 2.020 | 4.130 | cond1/cond2 | 0.857 | 0.217 | -0.611 | 0.541 |
|  | 2 | 3.370 | 0.594 | 2.390 | 4.760 | cond1/cond3 | NA | NA | NA | NA |
|  | 3 | NA | NA | NA | NA | cond2/cond3 | NA | NA | NA | NA |
| HAR | 1 | 7.500 | 1.200 | 5.490 | 10.250 | cond1/cond2 | 1.273 | 0.296 | 1.037 | 0.553 |
|  | 2 | 5.890 | 0.997 | 4.230 | 8.210 | cond1/cond3 | 0.710 | 0.172 | -1.410 | 0.336 |
|  | 3 | 10.560 | 1.940 | 7.380 | 15.130 | cond2/cond3 | 0.558 | 0.139 | -2.340 | 0.051 |
| FUY | 1 | 8.540 | 1.360 | 6.240 | 11.670 | cond1/cond2 | 1.435 | 0.329 | 1.574 | 0.257 |
|  | 2 | 5.950 | 0.981 | 4.300 | 8.220 | cond1/cond3 | 1.583 | 0.431 | 1.687 | 0.210 |
|  | 3 | 5.390 | 1.190 | 3.500 | 8.310 | cond2/cond3 | 1.103 | 0.304 | 0.356 | 0.932 |
| NAT | 1 | 8.100 | 1.320 | 5.890 | 11.130 | cond1/cond2 | 2.502 | 0.625 | 3.672 | 0.001 |
|  | 2 | 3.240 | 0.614 | 2.230 | 4.690 | cond1/cond3 | 0.996 | 0.233 | -0.019 | 0.999 |
|  | 3 | 8.130 | 1.370 | 5.850 | 11.310 | cond2/cond3 | 0.398 | 0.101 | -3.634 | 0.001 |

**Table S6.** Outcome of the GLM using a Tweedie distribution run with the interaction term between ID and Condition as a predictor. The durations of SB were the response variable and the observation time was included as an offset.

| **Term** | **Estimate** | **SE** | **Z value** | **p value** |
| --- | --- | --- | --- | --- |
| Intercept | -0.577 | 0.270 | -2.138 | 0.034 |
| HAR | -45.279 | <0.001 | -0.001 | 0.999 |
| MIT | -0.976 | 0.432 | -2.261 | 0.024 |
| NAT | -45.693 | <0.001 | -0.001 | 0.999 |
| cond2 | -6.837 | 1.544 | -4.429 | <0.001 |
| cond3 | -5.821 | 1.565 | -3.719 | <0.001 |
| HAR:cond2 | 8.499 | <0.001 | <0.001 | 1.000 |
| MIT:cond2 | 4.573 | <0.001 | 2.706 | 0.007 |
| NAT:cond2 | 8.035 | <0.001 | <0.001 | 1.000 |
| HAR:cond3 | 5.901 | <0.001 | <0.001 | 1.000 |
| NAT:cond3 | 6.745 | <0.001 | <0.001 | 1.000 |

**Table S6.1.** Outcome of the pairwise comparisons of estimated marginal means for the SB durations of each individual between Conditions, including contrast estimates. Tukey’s adjustment was used for multiple comparisons.

| **Estimated marginal means** | | | | | | **Contrasts** | | | | |
| --- | --- | --- | --- | --- | --- | --- | --- | --- | --- | --- |
| **ID** | **condition** | **response** | **SE** | **LCL** | **UCL** | **contrast** | **ratio** | **SE** | **Z** | **p value** |
| MIT | 1 | 1.364 | 0.460 | 0.705 | 3.000 | cond1/cond2 | 9.619 | 6.610 | 3.295 | 0.001 |
|  | 2 | 0.142 | 0.085 | 0.044 | 0.000 | cond1/cond3 | NA | NA | NA | NA |
|  | 3 | NA | NA | NA | NA | cond2/cond3 | NA | NA | NA | NA |
| HAR | 1 | 0.000 | 0.000 | 0.000 | 0.000 | cond1/cond2 | 0.190 | 0.009 | 0.000 | 1.000 |
|  | 2 | 0.000 | 0.000 | 0.000 | 0.000 | cond1/cond3 | 0.923 | <0.001 | 0.000 | 1.000 |
|  | 3 | 0.000 | 0.000 | 0.000 | 0.000 | cond2/cond3 | 4.864 | <0.001 | 0.000 | 1.000 |
| FUY | 1 | 3.621 | 0.977 | 2.134 | 6.000 | cond1/cond2 | 931.788 | 0.001 | 4.429 | <0.001 |
|  | 2 | 0.004 | 0.006 | <0.001 | 0.000 | cond1/cond3 | 337.452 | 0.052 | 3.719 | <0.001 |
|  | 3 | 0.011 | 0.017 | 0.001 | 0.000 | cond2/cond3 | 0.362 | 0.780 | -0.469 | 0.886 |
| NAT | 1 | 0.000 | 0.000 | 0.000 | 0.000 | cond1/cond2 | 0.302 | <0.001 | 0.000 | 1.000 |
|  | 2 | 0.000 | 0.000 | 0.000 | 0.000 | cond1/cond3 | 0.397 | <0.001 | 0.000 | 1.000 |
|  | 3 | 0.000 | 0.000 | 0.000 | 0.000 | cond2/cond3 | 1.315 | <0.001 | 0.000 | 1.000 |
